# Cognitive behaviour therapy for older adults experiencing insomnia and depression in a community mental health setting: Study protocol for a randomised controlled trial

**DOI:** 10.1186/s13063-015-1066-6

**Published:** 2015-11-27

**Authors:** Paul Sadler, Suzanne McLaren, Britt Klein, Megan Jenkins, Jack Harvey

**Affiliations:** School of Health Sciences & Psychology, Faculty of Health, Federation University Australia, PO Box 663, Ballarat, 3353 Victoria Australia; Centre for Biopsychosocial and eHealth Research & Innovation, Research & Innovation Portfolio, Faculty of Health, Collaborative Research Network Federation University Australia, Ballarat, Victoria Australia; National Institute for Mental Health Research, Australian National University, Canberra, ACT Australia

**Keywords:** Cognitive behaviour therapy, insomnia, depression, older adults, mental health services, randomised controlled trial

## Abstract

**Background:**

Cognitive behaviour therapy for insomnia (CBT-I) is a well-established treatment; however, the evidence is largely limited to homogenous samples. Although emerging research has indicated that CBT-I is also effective for comorbid insomnia, CBT-I has not been tested among a complex sample of older adults with comorbid insomnia and depression. Furthermore, no study has explored whether modifying CBT-I to target associated depressive symptoms could potentially enhance sleep and mood outcomes. Therefore, this study aims to report a protocol designed to test whether an advanced form of CBT for insomnia and depression (CBT-I-D) is more effective at reducing insomnia and depressive symptoms compared to a standard CBT-I and psychoeducation control group (PCG) for older adults in a community mental health setting.

**Methods/Design:**

We aim to recruit 150 older adults with comorbid insomnia who have presented to community mental health services for depression. Eligible participants will be randomly allocated via block/cluster randomisation to one of three group therapy conditions: CBT-I, CBT-I-D, or PCG. Participants who receive CBT-I will only practice strategies designed to improve their sleep, whereas participants who receive CBT-I-D will practice additional strategies designed to also improve their mood. This trial will implement a mixed-methods design involving quantitative outcome measures and qualitative focus groups. The primary outcome measures are insomnia and depression severity, and secondary outcomes are anxiety, hopelessness, beliefs about sleep, comorbid sleep conditions, and health. Outcomes will be assessed at pre-intervention (week 0), post-intervention (week 8), and 3-month follow-up (week 20).

**Discussion:**

This CBT study protocol has been designed to address comorbid insomnia and depression for older adults receiving community mental health services. The proposed trial will determine whether CBT-I is more effective for older adults with comorbid insomnia and depression compared to a PCG. It will also establish whether an advanced form of CBT-I-D generates greater reductions in insomnia and depression severity compared to standard CBT-I. The results from the proposed trial are anticipated to have important clinical implications for older adults, researchers, therapists, and community mental health services.

**Trial registration:**

Australian and New Zealand Clinical Trials Registry (ANZCTR): ACTRN: 12615000067572, Date Registered 12 December 2014.

## Background

Leaders in the field of cognitive behaviour therapy for insomnia (CBT-I) have suggested that this form of psychological treatment needs to be tested in more diverse samples and settings [8, 41, 71]. Promising research has indicated that CBT-I is effective at treating comorbid or secondary insomnia [17, 23, 35]; however, the effectiveness of CBT-I has not been examined among a complex sample of older adults receiving community mental health services for depression.

Insomnia and depression are commonly co-occurring conditions experienced by older adults [55]. Studies have reported that up to 90 % of individuals with depression report problems with comorbid insomnia [70]. Historically, insomnia was believed to have been primarily a symptom or natural consequence of depression [31]. More recent evidence has demonstrated that insomnia often precedes the onset of depression among older adults [48, 55] and can serve as an influential risk factor for depressive relapse [49]. Furthermore, studies have suggested that many of the symptoms required for the diagnosis of depression (for example, tiredness, amotivation, depressed mood, hopelessness, and poor concentration) can be attributed to increased levels of insomnia [31, 64]. Collectively, these findings have led researchers to hypothesise that, in some cases, the relation between insomnia and depression is reciprocal, as the two disorders can aggravate and maintain each other [8, 32]. Therefore, insomnia becomes not only a symptom of depression but can become an independent dysfunctional process and a comorbid disorder that can subsequently jeopardise depression treatment.

There is preliminary data that suggests CBT-I could have a positive effect on comorbid depression (for example, [28, 32, 72]). This body of research, however, is still in its infancy and contains several significant limitations that restrict the strength and generalisability of the findings (for example, younger participants, small sample sizes, homogenous groups, and no randomisation design or comparison/control group). Therefore, constructing a study protocol that investigates whether CBT-I is effective for older adults who are engaged with a mental health service for depression would significantly contribute to this body of literature.

No CBT-I trial has specifically investigated whether additional therapeutic modifications need to be considered when treating depression among older adults. Researchers have suggested this is an important area of future research because older adults with depression may require a more advanced treatment program than standard CBT-I alone [35, 61, 62]. The original CBT for depression manual [4] briefly discussed strategies to address symptoms of insomnia, most notably cognitively reframing negative predictions about poor sleep and increasing meaningful activities during the day. Integrating CBT for insomnia and depression (CBT-I-D) could be particularly helpful when staying in bed becomes an escape from the distress associated with the depression itself [61, 62]. This increased desire to sleep and withdraw from daily activities is likely to increase the attempts to sleep, which in turn, can exacerbate cognitive and somatic arousal and interfere with overall sleep quality [62]. Thus working simultaneously on both issues could create an increased therapeutic effect and further enhance sleep and mood outcomes. To date, no study has examined whether an advanced form of CBT-I-D produces greater reductions in insomnia and depression severity compared to CBT-I alone.

### Aims and hypotheses

The purpose of this study is to present a protocol that has been designed to evaluate whether CBT-I is effective for older adults with comorbid insomnia and depression in community mental health settings. This study aims to investigate whether there are significant differences between the three conditions (CBT-I, CBT-I-D, and PCG), with a particular focus on whether CBT-I-D produces greater reductions in insomnia and depression severity compared to CBT-I. The final aim of the study is to qualitatively explore the participants’ experience of taking part in the trial. This feedback will play an important role in improving future studies that explore the delivery of CBT-I for older adults with complex conditions.

There are two central hypotheses in this study. First, it is expected that participants who are randomised to the CBT-I condition will report a significant reduction in insomnia severity compared to participants who are randomised to the PCG condition at post (week 8) and follow-up (week 20) assessments. Second, it is hypothesised that participants who are randomised to the CBT-I-D condition will report greater reductions in insomnia and depression severity compared to participants who are randomised to the CBT-I and PCG conditions at post- and follow-up assessments.

## Methods/Design

### Trial design

**Fig. 1:**
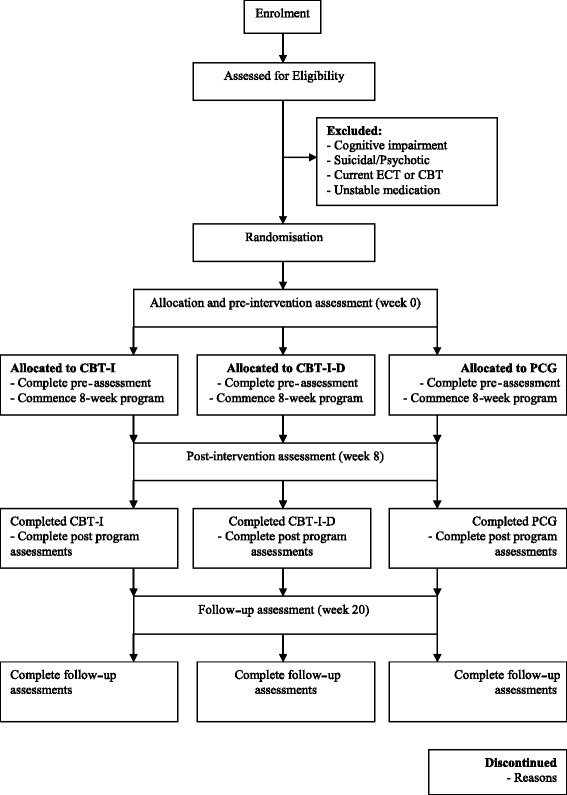
Study design and participant flow chart. Note: ECT = Electroconvulsive Therapy, CBT = Cognitive Behaviour Therapy, CBT-I = CBT for Insomnia, CBT-I-D = CBT for Insomnia and Depression, PCG = Psychoeducation Control Group

This trial will follow the International CONSORT (Consolidated Standards of Reporting Trials) guidelines and evaluate conditions using a randomised control trial (RCT) design [56]. Participants who are eligible for the trial will be randomly allocated to one of three conditions: CBT-I, CBT-I-D, or PCG. Outcomes will be assessed at pre-intervention (week 0), post-intervention (week 8), and 3-month follow-up (week 20). Figure 1 illustrates the study’s design and expected participant flow chart.

### Participants

We are recruiting participants through an aged persons’ community mental health services in regional catchments of Victoria, Australia. A highly inclusive approach will be used to maximise participation and increase generalisability. Participants will be eligible for the trial if they meet the following criteria: aged 65 years or above, been referred to an aged persons’ mental health service, current Comorbid Insomnia Disorder [1], and past and/or current Major Depressive Disorder [1].

Participants who meet criteria for Comorbid Insomnia Disorder will report (a) dissatisfaction with their sleep associated with either difficulty initiating sleep, difficulty maintaining sleep, or early morning awakening with the inability to return to sleep; (b) causing significant impairment of functioning; (c) at least 3 nights per week; and (d) is present for at least 3 months. Participants who meet criteria for past and/or current Major Depressive Disorder will report (a) depressed mood or lack of interest/pleasure consistently over a 2-week period; (b) five or more of the following symptoms, which include change of appetite/weight, sleep disturbance, psychomotor agitation/retardation, loss of energy, worthlessness/guilt, indecisiveness, or suicide ideation; and (c) the symptoms cause significant functional impairment.

Participants will be excluded if they are cognitively impaired (Mini Mental State Exam score below 24; [21]); in a crisis stage of mental illness (for example, exhibiting psychotic features or demonstrating active suicidal intent/plan); on unstable doses of medication (this means eligible participants will need to be on the same doses of their prescribed medications for at least 1 month prior to commencing the intervention); currently participating in Electro-Convulsive Therapy (ECT); or currently participating in CBT with another psychotherapist.

### Assessment

Potential participants will be provided with a trial invitation via the aged persons’ mental health service staff. Interested participants will be asked to sign an informed consent document prior to commencing the eligibility assessment. Once consent is obtained, participants will be invited to participate in a preliminary insomnia screen. This screen will correspond with the *Diagnostic and Statistical Manual for Mental Disorders 5*^*th*^*edition (DSM-V)* criteria for Comorbid Insomnia Disorder [1] and follow Morin and Benca’s [40] insomnia assessment guidelines. Participants meeting comorbid insomnia criteria will then be invited to participate in a clinical interview eligibility assessment.

Demographic information will be collected, including age, sex, relationship status, education level, income source, accommodation type, religious beliefs, past occupation, medical history, and medications. Participants will be asked if they have been diagnosed with a sleep disorder, and if so, their treatment (for example, CPAP) and perceived treatment effectiveness. Participants will also be screened for sleep apnea and other comorbid sleep conditions (for example, restless legs) by administering the SLEEP-50 [65]. It is important to note that participants with comorbid medical, sleep, and psychiatric conditions will be included in this trial.

The Mini International Neuropsychiatric Interview (MINI 6.0; [58, 59]) will be administered to assess Major Depressive Disorder (past/current episode) and other high prevalence mental disorders. The MINI is a clinical psychiatric diagnostic tool with structured ‘yes’ or ‘no’ responses, (for example, ‘Were you ever depressed or down, most of the day, nearly every day, for two weeks?’). The MINI is more efficient to administer compared to other structured diagnostic instruments (for example, SCID), taking approximately 15 to 30 minutes to complete. The MINI corresponds with the *DSM-IV* diagnostic criteria, which is commonly applied in research settings. The MINI has demonstrated inter-rater and test-retest reliability and validity across diverse populations (for example, [58, 60]) including older adults [51]. Since the MINI was standardised on the *DSM-IV*, the principal investigator updated the items for each disorder to be consistent with the *DSM-V* criteria.

The Mini-Mental State Examination (MMSE; [21]) will be used to screen for possible cognitive impairment. The MMSE contains 30 items that assesses a range of cognitive functions, such as orientation, short-term memory, language, comprehension, attention and calculation [21]. Total scores on the MMSE range from 0 to 30. Participants with scores of 23 or below are considered likely to have a cognitive impairment [21]. The MMSE has shown high levels of reliability and validity; for instance, studies have reported internal consistencies of up to Cronbach’s α = .90, and test-retest reliabilities over a 24-hour period above *r* = .85 [36]. In addition, Mitchell [36] found the MMSE demonstrated adequate sensitivity (71.1 % to 85.1 %) and specificity (81.3 % to 95.6 %) across a range of settings.

### Measures

Once the eligible clinical assessment has been completed, eligible participants will be asked to complete a self-report questionnaire package at pre-treatment (week 0) to collect baseline outcome measures. Participants will also be asked to complete the outcome measures at post-intervention (week 8) and 3-month follow-up (week 20). Participants will be financially reimbursed ($20) each time they complete the questionnaire pack ($60 total) to acknowledge this additional time dedicated toward the study. Table 1 shows the schedule and frequency of the assessments during the trial.

**Table 1:** Assessments and administration frequency

Concept	Measure	Instrument	Conditions		
			Week	Week	Week
			0	8	20
Primary outcomes	Insomnia severity	ISI	x	x	x
	Depression severity	GDS	x	x	x
Secondary outcomes	Sleep quality	CSD	x	x	x
	Diagnosis	MINI 6.0	x	x	x
	Anxiety	GAI-SF	x	x	x
	Hopelessness	BHS	x	x	x
	Beliefs about sleep	DBAS-10	x	x	x
	Sleep conditions	SLEEP-50	x	x	x
	Health	EQ-5D-3 L	x	x	x
Eligibility	Insomnia diagnosis	DSM-V Insomnia Screen	x	x	x
	Cognitive screen	MMSE	x	-	-
Moderators	Demographics	Demographic Information	x	-	-
Treatment	Expectations	TCI	x	-	-
	Feedback	Reflective Focus Group	-	x	-

*Note: ISI* Insomnia Severity Index, *CSD* Consensus Sleep Diary, *GDS* Geriatric Depression Scale, *MINI* Mini International Neuropsychiatric Interview, *GAI-SF* Geriatric Anxiety Inventory Short Form, *BHS* Beck Hopelessness Scale, *DBAS-10* Dysfunctional Beliefs and Attitudes About Sleep 10-Item Scale, *EQ-5D-3 L* EuroQol Health Scale, *MMSE* Mini Mental State Examination, *TCI* Treatment Credibility Index

The Insomnia Severity Index (ISI; [38]) is a self-report instrument measuring the participant’s perception of his or her level of insomnia. The ISI assesses the subjective symptoms and consequences of insomnia, as well as the degree of concerns or distress caused by those difficulties during the previous two weeks. The ISI comprises seven items assessing the severity of sleep-onset and sleep maintenance difficulties (both nocturnal and early morning awakenings), satisfaction with current sleep pattern, interference with daily functioning, ability to notice the level of sleep impairment, and degree of distress or concern caused by the sleep problem. Each item is rated on a five-point scale (0 = none, to 4 = very). Total scores range from 0 to 28, with higher scores representing more severe levels of insomnia. Total scores of 0 to 7 indicate no clinically significant insomnia; scores of 8 to 14 indicate sub-threshold insomnia; and scores of 15 to 28 indicate moderate to severe levels of insomnia. Bastein et al. [2] conducted a psychometric study of the ISI among older adults. It was found that the mean item-total correlations were *r* = .56 at pre-treatment, *r* = .69 at post-treatment, and *r* = .72 at follow-up. The internal reliability coefficients remained stable from .76 at baseline to .78 at follow-up. Bastein et al. concluded that the ISI was a useful clinical tool for screening insomnia severity among older adults or as an outcome measure in insomnia treatment research.

The Consensus Sleep Diary (CSD; [12]) allows individuals to record information about their nightly sleep pattern. A recent panel of international sleep experts [12] developed the CSD as the current standardised sleep diary measure for insomnia research. The CSD contains nine items that were considered by the expert panel to represent the most critical sleep parameters (for example, ‘What time did you get into bed?’). The CSD was formatted so that 1 week of nightly sleep data could be recorded on a single diary page. The CSD instructions included general information, such as what to do if the respondent misses recording on a particular day, and an item-by-item instruction guide to enhance the likelihood of correct item interpretation. Additional instructions indicate that all items should be completed in the morning within 1 hour of getting out of bed. Previous research (for example, [52]) has reported that sleep diaries were accurate when compared to polysomnographic data (kappa = .87; sensitivity = 92.3 %; specificity, 95.6 %). Researchers agree that having individuals prospectively self-monitor their sleep with a sleep diary is a useful psychometric tool for insomnia assessment and for examining treatment effects (for example, [10]).

The SLEEP-50 [65] is designed to measure the intensity of an individual’s subjective sleep complaints on a range of sleep conditions, including sleep apnea, insomnia, narcolepsy, restless legs, circadian rhythms, sleepwalking, nightmares, other factors influencing sleep, and functioning impairment. The SLEEP-50 is scored on a four-point Likert scale from 1 ‘not at all’ to 4 ‘very much’. An example sleep apnea item includes ‘I am told that I hold my breath when sleeping’. The SLEEP-50 is considered a reliable and valid screening tool for comorbid sleep conditions, Spoormaker et al. reporting a 3-week test-retest reliability of *r* = .78 and Cronbach’s α of .85.

The Dysfunctional Beliefs and Attitudes About Sleep 10-Item Scale (DBAS-10; [19]) measures the intensity of maladaptive beliefs about sleep (for example ‘When I have trouble getting to sleep, I should stay in bed and try harder’). Espie et al. redeveloped this measure into a shorter version of 10 items from the original 30-item DBAS scale of Morin [38]. Participants complete each question using a 10-cm visual analogue scale, anchored with strongly disagree and strongly agree. Added together, the 10 item responses provide the final DBAS score. Total scores range from 0 to 100, with higher scores representing more rigid or stronger levels of dysfunctional beliefs and attitudes about sleep. The DBAS-10 was has been found be a reliable measure of dysfunctional beliefs about sleep among older adults (Cronbach’s *α* = 0.88; [55]).

The Geriatric Depression Scale (GDS; [74]) is a 30-item clinician-rated questionnaire used to assess depression severity specifically for older adults. Participants are asked to respond ‘yes/no’ to each item, for example, ‘Over the past week have you felt that your life has been empty?’ Possible scores range from 0 to 30, with higher scores indicating the presence of more depressive symptomatology. A total score of 0 to 9 indicates normal levels of depression, 10 to 19 indicates mild to moderate levels of depression, and 20 to 30 indicates severe depression [74]. Yesavage et al. reported that the GDS had a high degree of internal consistency (Cronbach’s α = .94) and weekly test-retest reliability (*r* = .85), and displayed strong correlations with other well-validated depression measures (for example, Hamilton Rating Scale for Depression, *r* = .83).

The Geriatric Anxiety Inventory Short Form (GAI-SF; [11]) is a five-item version of the original 20-item Geriatric Anxiety Inventory [46], which assesses anxiety severity among older adults. Respondents answer yes/no to the five items, for example, ‘I worry a lot of the time’. A score of three or more indicates probable anxiety disorder. At this cut off, sensitivity was 75 %, and specificity was 87 %. Internal consistency was also found to be high, Cronbach’s α at .81 [11].

The Beck Hopelessness Scale (BHS; [5]) is a self-report instrument, which entails 20 true/false statements designed to assess the degree to which an individual holds over negative beliefs about the future from the previous week. For example, a participant that answers ‘True’ to the follow question, ‘My future seems dark to me’, would score 1 point and represent a pessimistic response. Each of the 20 statements is scored 0 or 1, with the total being calculated by summing the pessimistic responses for the 20 items. The total BHS score ranges from 0 to 20, with higher scores reflecting higher levels of hopelessness. A total score ranging from 0 to 3 identifies minimal hopelessness, from 4 to 8 identifies mild hopelessness, from 9 to 14 identifies moderate hopelessness, and greater than 14 identifies severe hopelessness. The BHS has been used widely among older adult community samples (for example, [68]). Test-retest reliability of the BHS over a 6-week period ranged from *r* = .66 to *r* = .69, and internal consistency was high, Cronbach’s α = .93 [7].

The EQ-5D-3 L scale [69] measures perceived health status. It comprises five items measuring mobility, self-care, usual activities, pain, and mood. Each item is rated on a three-point Likert scale (1 = no problems, 2 = some problems, 3 = extreme problems). The EQ-5D-3 L also contains an additional item, which asks respondents to rate their overall current health level using a visual analogue scale from ‘0 = worst imaginable health’ to ‘100 = best imaginable health’. The EQ-5D-3 L has been validated across several populations and countries (for example, [24]) and is considered an appropriate outcome measure of health status [9].

The Treatment Credibility Index (TCI; [15]) measures treatment credibility and expectancy for use in clinical trials. It involves six items that cover two factors: thinking-based credibility (for example, ‘How logical does the therapy offered to you seem?’) and feeling-based expectancy (for example, ‘How much improvement in your symptoms do you really feel will occur?’). The TCI demonstrated high internal consistency (Cronbach’s α = .85) and good test-retest reliability (*r* = .82 for expectancy and *r* = .75 for credibility).

### Outcome assessors

The post- and follow-up outcome assessments will be conducted by provisional and general registered psychologists who are on clinical placement or employed with the aged persons’ mental health services. The independent assessors will have no therapeutic involvement with the intervention group they are testing. They will also be blinded to the condition they are evaluating. The assessors will receive training and supervision by the principal investigator throughout the trial.

### Primary outcomes

The primary outcomes being assessed are insomnia and depression severity. Participants’ level of insomnia (ISI; [38]) and depression (GDS; [74]) will be assessed using validated self-report questionnaires.

### Secondary outcomes

The secondary outcomes being assessed are sleep quality (CSD; [12]), mental health diagnosis (MINI 6.0; [58]), anxiety (GAI-SF; [11]), hopelessness (BHS; [4]), beliefs/attitudes about sleep (DBAS-10; [38]), comorbid sleep conditions (SLEEP-50; [65]), and health status (EQ-5D-3 L; [69]).

### Interventions

#### Cognitive Behaviour Therapy for Insomnia (CBT-I)

CBT-I is a structured, time limited, multi-component program that includes a combination of educational, cognitive, and behavioural interventions (for example, [3, 34, 38, 40]). The main objective of CBT-I is to change factors that perpetuate insomnia, including behavioural factors (poor sleep habits and irregular sleep schedules), psychological factors (unrealistic expectations about sleep and unhelpful sleep beliefs), and physiological factors (somatic tension and cognitive hyperarousal). The CBT-I program will closely follow Morin’s CBT-I treatment guidelines (for example, [3, 38–40, 42]), and will also acknowledge the works from Lichstein (for example, [29]) and Rybarczyk (for example, [53]) in treating comorbid insomnia.

Participants assigned to the CBT-I condition will attend eight weekly 60 to 90 minute sessions, which will include small groups comprising five to six participants. Group therapy was chosen instead of individual therapy because working in groups is more cost-effective and time-efficient [27]. This is particularly important for regionally based community mental health settings where resources can be limited [54]. Group therapy also creates an opportunity to increase participants’ sense of belonging, provide peer-to-peer support and motivation [27]. The format and delivery of treatment will follow a clear CBT session structure [29, 40]. Information handouts and homework worksheets for each CBT-I intervention will be provided and kept in a daily workbook. The principal investigator of this study carefully designed these worksheets to be easily legible and workable for older adults. Participants will be educated about the strong relationship between homework compliance, group connectedness, attendance, and treatment effect [18, 26]. Each participant will also be encouraged to have a supportive person (for example, family member or friend) to assist their homework compliance during the trial [26].

The behavioural sleep interventions of CBT-I will be introduced in the first four sessions of the program and include stimulus control/restriction, sleep hygiene, and relaxation skills [38, 40, 42]. The cognitive sleep interventions in CBT-I will be covered in the latter half of the program, which includes cognitive restructuring. During this second half of the program, considerable discussion will be devoted to relapse prevention and maintaining progress [40, 42]. Table 2 illustrates a summary of the session interventions for each condition.

**Table 2:** Summary of session interventions

Session	CBT-I	CBT-I-D	PCG
1	Introduction	Introduction	Introduction
2	Stimulus control	Stimulus control	Insomnia
	Sleep restriction	Sleep restriction	
3	Sleep hygiene	Sleep hygiene	Sleep health
		Behavioural activation	
4	Relaxation	Relaxation	Sleep and mood
		Behavioural activation	
5	Relaxation	Relaxation	Sleep and mood
	Cognitive reframing (insomnia)	Cognitive reframing (insomnia)	
6	Cognitive reframing (insomnia)	Cognitive reframing (insomnia/depression)	Beliefs about sleep
7	Cognitive reframing (insomnia)	Cognitive reframing (depression)	Beliefs about sleep
		Affirmations	
8	Relapse prevention	Affirmations	Summary
		Relapse prevention	

*Note: CBT-I* Cognitive Behaviour Therapy for Insomnia, *CBT-I-D* Cognitive Behaviour Therapy for Insomnia and Depression, *PCG* Psychoeducation Control Group

#### Cognitive Behaviour Therapy for Insomnia and Depression (CBT-I-D)

Participants who are assigned to the CBT-I-D group will complete the same program as the participants assigned to the CBT-I group (for example, eight sessions of CBT-I); however, the CBT-I-D group will include an additional three CBT strategies to address comorbid depression (behavioural activation, cognitive reframing for depression, and affirmations).

The first additional intervention in this group will include behavioural activation. Behavioural activation, or activity scheduling, is a common intervention included in CBT for depression, which aims to increase an individual’s level of activity, socialisation, and sense of achievement/pleasure [6, 73, 75]. This involves setting behavioural goals and planned activities, and keeping a daily activity schedule to explore how the participants’ feel before and after an activity. These behavioural experiments will be regularly discussed and monitored during the program.

The second and third additional elements of CBT-I-D involve including a broader focus on cognitive reframing and positive affirmations. In addition to reframing sleep-related dysfunctional beliefs, participants in this group will also learn to reframe important negative thoughts related to their depression (for example, [4, 6, 73]). This will be achieved through additional thought records and positive affirmation hope cards introduced during the latter four sessions of the program.

### Control

#### Psycho-Education Control Group (PCG)

Participants assigned to the PCG will also attend eight weekly, 60 to 90 minute sessions, in small groups comprising five to six participants. The content of these sessions will include psychoeducation about sleep, insomnia, and depression, with no active cognitive or behavioural change strategies (for example, instructions, guidelines, homework tasks, relapse prevention). This group will take a more supportive approach rather than a structured CBT format. Morin et al. [41] and Mogenthaler et al. [44] reported that providing only psycho-education is an appropriate control condition in insomnia treatment research.

### Therapists

The therapists who co-facilitate the groups will be provisionally registered psychologists who are completing their final clinical psychology placement within the aged persons’ mental health services. The groups will be co-facilitated due to the heterogeneous and complex nature of this sample. The therapists will receive daily training and clinical supervision throughout the trial by the principal investigator.

### Focus groups

At the end of the final session (Session 8) of each condition, participants will be invited to participate in a focus group to reflect on their experiences from participating in the trial. They will be asked eight questions (for example, ‘What specific strategy was most helpful during the program?’ that have been designed to inform future CBT programs in this field. The focus groups will be conducted by the principal investigator and audio recorded for qualitative statistical analysis. The principal investigator will approach the focus groups in a non-judgemental and curious manner, encouraging open discussion and reflection rather than challenging the participants’ responses [50].

### Randomisation

The randomisation allocation process of participants to treatment groups will comply with CONSORT guidelines (for example, [37]). A block/cluster randomisation design utilising random permuted blocks with randomised block sizes will be implemented to randomly allocate each participant group to one of three conditions throughout the trial. Block randomisation is commonly used in small- to moderate-sized RCTs to ensure that approximately equal numbers of participant groups in the clustered context are allocated to each condition [67]. The computer-generated random allocations will be generated by an independent researcher at Federation University Australia and will be stored on a password-protected computer back-up to a secure university file server. Random allocation will occur after the pre-treatment assessments have been completed to ensure eligibility has been met. Each successive random allocation will be communicated to the principal investigator only when five to six eligible participants are ready to commence a group. Participants will be unaware of the study’s hypotheses or which group they have been randomly allocated. The co-therapists will not be blinded to the treatment conditions.

### Sample size calculation

Statistical power analysis was based on an examination of treatment-time interactions in a repeated measures analysis of covariance (RMANCOVA) of the ISI measure [38], with adjustment for confounders. We specified an initial value of 18 points in the adjusted mean of the ISI score at pre-intervention [22]; a mean reduction of 10 points from pre- to post-intervention for the CBT-I treatment [22]; and a target value of two points difference between the pre-post changes for each of the three treatment conditions (that is, -8, −10, and −12 points, respectively) [43], with no further change at follow-up [43], and assuming a ‘within-treatments’ SD of 3.4 points [22]. This resulted in an effect size of 0.23. Under the assumptions of constant correlation over time (sphericity), with an estimated magnitude of *r* = 0.6 based on reported test-retest reliabilities of 0.79 [14] and 0.86 [76], and allowance for a design effect [16] of 1.07 due to clustering of participants within treatment groups (based on an assumed cluster size of five and an intra-class correlation of 0.017, [22]), with a significance level of 5 % and 80 % power, the required sample size calculated using GPower software [20] is *N* = 35 (33 × 1.07). Maintaining the same specifications, but focusing solely on the CBT-I and CBT-I-D conditions, the specific target difference of two points between the pre-post changes for these two particular treatments corresponds to an effect size of 0.13. In order to have 80 % power to detect this particular difference in a post hoc pairwise comparison, the required sample size is increased to *N* = 85 (78 × 1.07).

With regard to the GDS measure [74], Secker and Brown [57] reported pre- and post-intervention means of 7.0 and 4.4 in a treatment group, and 5.8 and 4.7 in a control group, a difference of 1.5 units [(7.0 - 4.4 = 2.6) - (5.8 - 4.7 = 1.1)] in the pre- and post-change, and an average within-treatment *SD* of 3.5. Replacing the values of 2.0 and 3.4 in the ISI analysis with 1.5 and 3.5 results in effect sizes of 0.16 with respect to comparisons across the three conditions and 0.10 with respect to comparisons between the two conditions. This leads to required sample sizes of *N* = 68 with respect to comparison of mean GDS across the three conditions, and *N* = 150 with respect to comparison of mean GDS between two of the conditions. Because this is larger than *N* = 85 from the ISI projection, *N* = 150 becomes our target sample size, which equates to 50 participants or 10 groups per condition.

### Statistical analyses

Data will be entered, screened, and analysed using SPSS Version 21 [25]. A RMANCOVA will be implemented to explore differences between the conditions over time, and calculate effect sizes to investigate the treatment effect. An intention-to-treat methodology will be applied via multiple imputation. The qualitative data obtained from the focus groups will be analysed according to the Interpretative Phenomenological Analysis (IPA) method (for example, [50, 63]). This will be achieved by transcribing the data verbatim from audio recordings into text. The text transcripts will be read several times to extract emerging codes, which will then be transformed into themes [50]. Themes are likely to identify both something of importance to the participants, and also convey a meaning of their reflection [63]. These themes will form the basis for the qualitative results. Direct quotes from the participants will also be reported to highlight the thematic findings [63].

### Ethics approval and trial registration

This trial will be conducted in accordance with the ethical guidelines outlined in the National Statement on Ethical Conduct in Human Research [45]. Ethics approval for this project has been granted by the Federation University Australia Human Research Ethics Committee (E-14-042), the Latrobe Regional Hospital Human Research Ethics Committee (2014-02-LNR), and the Peninsula Health Human Research Ethics Committee (HREC-15-PH-4). The trial has also been registered with the Australian New Zealand Clinical Trial Registry, ACTRN: 12615000067572.

## Discussion

This paper describes the study protocol for the development and evaluation of a CBT program for older adults with comorbid insomnia and depression. The results from this trial will represent a significant step forward in this field and assist in advancing CBT-I for older adults receiving mental health services for depression. Several important clinical implications could result from this project.

One of the primary aims is to evaluate whether CBT-I is effective among a complex sample of older adults with comorbid insomnia and depression in a community mental health setting. We predict that CBT-I will be more effective at reducing insomnia severity compared to the PCG. If this hypothesis is supported, it will demonstrate that CBT-I is helpful at reducing insomnia severity for older adults with depression in a community mental health setting. This evidence will add to a growing research base that suggests CBT-I needs to be considered as a treatment option for individuals with comorbid insomnia [30, 66, 71].

Since previous research has suggested that comorbid insomnia is often misunderstood and inadequately treated [18, 40, 62], education and training play a vital role to address this disparity between research and clinical practice. This could involve providing workshops to mental health practitioners to raise their awareness of addressing comorbid insomnia during the assessment, case conceptualisation, and treatment planning phases. To ensure older adults with comorbid insomnia have access to this form of psychological treatment, mental health services could also consider training a therapist on their multidisciplinary team in CBT-I, or at least source an external CBT sleep specialist as a referral option. These implications could potentially improve therapeutic outcomes for older adults receiving mental health services by ensuring clinicians are better equipped to address comorbid insomnia.

This project also plans to explore whether improving an older adult’s sleep pattern with CBT-I has an associated positive impact on their mood. Recent preliminary evidence suggests CBT-I can have a positive effect of depression levels [28, 32, 72]; however, these studies have substantial methodological limitations that restrict the generalisability of the results. Consequently, this study aims to test whether CBT-I generates greater reductions in depressive severity compared to a PCG. If the results indicate that CBT-I is more effective at improving depression compared to the control condition, aged psychiatric services could consider adding CBT-I as a regular treatment option for their clients presenting with comorbid insomnia and depression.

A unique feature of this trial involves testing an advanced form of CBT-I that includes additional strategies to address comorbid depression among older adults. Although recent studies (for example, [71]) have suggested that specifically designed CBT-I programs may produce better overall outcomes for targeted populations (for example, older adults with comorbid insomnia and depression), no study has tested whether an advanced CBT-I-D program is more effective than a standard CBT-I program. The results following this specific investigation could highlight that CBT-I is sufficient to address both insomnia and depression or indicate CBT-I-D is clinically indicated to better treat these conditions simultaneously. This result could guide future CBT-I treatment programs in relation to either broadening or lessening the scope of treatment.

Finally, we are interested in studying the participants’ experiences from taking part in a CBT trial for insomnia and depression. The information gained from a mixed methods design will provide an opportunity to gather rich, personal data through older adults that can either confirm or disconfirm the questionnaire outcome measures [13, 33]. This can open new avenues of explanation for particular findings that traditional quantitative measures cannot detect [47]. For example, results from the reflective focus groups may indicate participants benefitted from specific CBT strategies that would not be revealed through quantitative data [33]. In addition, participants may provide innovative feedback that is particularly relevant to the delivery of CBT-I among this complex aged population. Therefore, it is expected that the qualitative findings from this project will enhance the direction of future research in this field.

Despite the important implications that may result from this study protocol, the project will likely include limitations. First, no physiological measure of sleep (for example, polysomnography) will be assessed to cross-validate the clinical interview and self-report data. This means that the assessments throughout the trial will be reliant on subjective measures from the participants and researchers, as opposed to objective biological data. Since no standardised clinical diagnostic instrument has been established yet for the *DSM-V*, the authors updated existing *DSM-IV* diagnostic instruments (for example, MINI 6.0) to be consistent with the *DSM-V* diagnostic criteria. Finally, participant recruitment and retention rates could be negatively affected due to this population likely experiencing stronger symptoms of tiredness, hopelessness, and amotivation. Hence, recruiting participants and keeping them engaged throughout the program will likely create significant challenges. Notwithstanding these limitations, the results following this study protocol will likely contribute important knowledge that improves case formulation and treatment planning for older adults with comorbid insomnia and depression.

## Trial status

Recruitment began in August 2014 and will continue until June 2016.

## Abbreviations

CBT: cognitive behaviour therapy
CBT-I: cognitive behaviour therapy for insomnia
CBT-I-D: cognitive behaviour therapy for insomnia and depression
PCG: psycho-education control group
RCT: randomised controlled trial
